# Analysis of the Interior Microclimate in Art Nouveau Heritage Buildings for the Protection of Exhibits and Human Health

**DOI:** 10.3390/ijerph192416599

**Published:** 2022-12-10

**Authors:** Alexandru Ilieș, Tudor Caciora, Florin Marcu, Zharas Berdenov, Gabriela Ilieș, Bahodirhon Safarov, Nicolaie Hodor, Vasile Grama, Maisa Ali Al Shomali, Dorina Camelia Ilies, Ovidiu Gaceu, Monica Costea, Damiannah Kieti

**Affiliations:** 1 Department of Geography, Tourism and Territorial Planning, Faculty of Geography, Tourism and Sport, University of Oradea, 1 Universitatii Street, 410087 Oradea, Romania; 2 Faculty of Medicine and Pharmacy, University of Oradea, 10 Piata, 1 Decembrie Street, 410073 Oradea, Romania; 3 Faculty of Science, L.N. Gumilyov Eurasian National University, 2 Satpayev Street, Nur-Sultan 010008, Kazakhstan; 4 Faculty of Geography, Babes-Bolyai University, Sighetu Marmatiei Extension, 6 Avram Iancu Street, 435500 Sighetu Marmatiei, Romania; 5 Department of Digital Economy, Samarkand State University, Samarkand 140104, Uzbekistan; 6 Faculty of Geography, Babes-Bolyai University, 5-6 Clinicilor Street, 400006 Cluj-Napoca, Romania; 7 Faculty of Engineering, Al-Balqa Applied University, P.O. Box 15008, Marka 11134, Jordan; 8 Faculty of Environmental Protection, University of Oradea, Gen Magheru Street, 410048 Oradea, Romania; 9 School of Tourism, Hospitality & Events Management, Department of Tourism, Moi University, Kesses, Eldoret 3900-30100, Kenya

**Keywords:** indoor air quality, cultural heritage, museum microclimate, preventive conservation, human health, risk assessment, pollutants

## Abstract

Poor air quality inside museums can have a double effect; on the one hand, influencing the integrity of the exhibits and on the other hand, endangering the health of employees and visitors. Both components can be very sensitive to the influence of the internal microclimate, therefore careful monitoring of the physical parameters and pollutants is required in order to maintain them within strict limits and thus to reduce the hazards that can be induced. The current study considers the determination and analysis of 15 indicators of the internal microclimate in an Art Nouveau museum built at the beginning of the 20th century in the Municipality of Oradea, Romania. The monitoring spanned a period of seven months, between September 2021 and March 2022, targeting three rooms of the museum with different characteristics and containing exhibits with a high degree of fragility. The results show that, although there are numerous indicators that have exceeded the thresholds induced by international standards, the possible negative impact on the exhibits and/or on human health remains moderate. This is due to the fact that, most of the time, exceeding the permitted limits are small or only sporadic, the values quickly returning to the permitted limits. Thus, only 22 of the 212 days of monitoring recorded marginal conditions regarding the quality of the indoor air, the rest having acceptable and good conditions. To improve the indoor conditions, a more careful management is needed, especially regarding the values of temperature, humidity, particulate matters, natural and artificial light, volatile organic compounds (VOC) and formaldehyde (HCHO), which during the measurements recorded high values that fluctuated in a wide spectrum. The obtained results can represent the basis for the development and implementation of long-term strategies for stabilizing the microclimatic conditions in the museum in order to preserve the exhibits preventively and to ensure a clean and safe environment for people.

## 1. Introduction

For centuries, museums and exhibitions have played an integral role in preserving the cultural heritage of societies, telling modern and ancient stories that could be forgotten about nations, communities and cultures [1,2,3,4]. Considering the great artistic and historical value of the exhibits from museums, it is a priority to protect their physical integrity in order to present them to the interested public in the best possible form [5]. One of the main elements that damage museum exhibits is the internal microclimate in the exhibition and storage rooms. Different indicators related to air quality (temperature, humidity, brightness, pollutants, chemical compounds, etc.), both of natural and anthropogenic origin, can have negative effects on the various materials from which the exhibits are composed, leading to their irreversible damage [6,7,8,9]. Over time, this subject aroused the curiosity of many researchers, who considered monitoring the internal microclimate in museums in an effort to limit the negative effects on the exhibits [10,11,12,13]. Following these studies, researchers suggested preventive conservation as a way to slow down the degradation process in museums. This involves monitoring, evaluating, and controlling the interior environmental conditions and maintaining them within strict limits [14,15,16,17].

In addition to having the potential to negatively impact the integrity of the exhibits, poor air quality inside museums can also have an impact on people’s health, as shown in the studies undertaken by different authors [18,19,20,21,22,23]. According to the U.S. Environmental Protection Agency (EPA) [24], pollutants identified in the indoor microclimate are among the top five risks factors for human health, while the World Health Organization (WHO) indicates that 2.7% of annual illnesses globally are due to low quality of the microclimate inside buildings [25].

Cincinelli et al. [26] and González-Martín et al. [27] consider that the most affected are museum employees and restaurateurs, who spend a lot of time in the indoor environment, the symptoms they can feel being respiratory illnesses, allergies and even cancerous diseases; poor air quality can also lead to a decrease in their productivity. At the same time, museum visitors are also likely to be affected by indoor air quality, even if they spend a rather short time in the indoor environment [28,29]. They are prone to ailments such as headaches, skin and eye irritations, repeated coughing and sneezing, dizziness and vomiting, severe fatigue, etc. [30,31,32,33].

The human component, besides the fact that it is very sensitive to indoor pollutants, can itself represent a source of microclimate pollution [5]. Thus, a large number of museum visitors can cause large increases in the concentration of CO_2_, a gas accumulated in the presence of people, or the different artificial solvents originating in perfumes or new clothes can be a source of microclimate pollution, affecting the total volume of volatile organic compounds (TVOC) in the air. A large number of visitors also bring a large volume of particles matter (PM) from the outdoor environment to the indoor environment, contributing to the volume of deployed particle matters (DPM) by dislocating the existing dust, which is then placed on the exhibits and inhaled by people [34,35,36]. In this sense, as indicated by Schieweck and Salthammer [37], it is important to find a consensus, to ensure the employees and visitors of the museum a healthy environment to carry out their activity (without restrictions), as well as to protect the exhibits against damage.

Numerous studies have examined the physical factors, as air temperature (T), brightness and relative humidity (RH), which have recognized effects on the integrity of artefacts and which call for close monitoring [38,39,40]. The two indicators’ excessive seasonal or daily volatility might eventually cause the cultural heritage artifacts to be irreparably damaged. T and RH values should be kept as close to the prescribed range as possible in order to prevent this. Therefore, they must not fluctuate significantly or frequently, and they must not record values that might injure exhibits and humans [41]. One of the primary causes of exhibit damage and adverse health consequences in humans could also be the cumulative impact of microclimatic variables and different pollutants [42,43].

Among the pollutants, some have harmful potential only on human health, such as CO_2_, CO, CH_4_ and too small or too large amounts of O_2_ [5,44], while other pollutants can be dangerous both for human health and for the integrity of museum exhibits. Particulate matter (PM) can induce disease symptoms in humans, through inhalation and can also cause aesthetic degradation of different materials (textiles being the most sensitive), especially if calcite accumulations are present [45,46]. High concentrations of VOCs, depending on the organic compounds contained, can cause various degradations of objects, as well as health problems for people; among these, HCHO, together with strong oxidants (such as O_3_), can oxidize into AcOH right on the surface of the objects [47,48]. AcOH is known to affect metals, glass, cellulose, pigments in paintings, etc. [49,50]. NO, NO_2_ and SO_2_ in the presence of water and different metals oxidize into H_2_SO_4_, which can induce major degradation of exhibits made of different metals, cellulose, leather, silk and textiles [50,51,52,53]. O_3_ is a strong oxidant, which reacts with organic materials and produces brittleness, fading and cracking [54], while H_2_S plays an important role in the deterioration of silver, copper, bronze and various pigments, producing corrosion [50,55].

In order to maintain an internal microclimate as clean as possible, it is necessary that the concentrations of physical variables and pollutants are kept within normal limits. These take into account the international standards in force, provided by the World Health Organization (WHO) or by various organizations and institutions that consider the establishment of thresholds for indicators that have the potential to harm the integrity of museum exhibits and human health. Even if some indicators only target human health, while others are dangerous for exhibits, in order to maintain an optimal internal microclimate in museums, a complete set of standards and guidelines must be considered [7].

**Figure 1:**
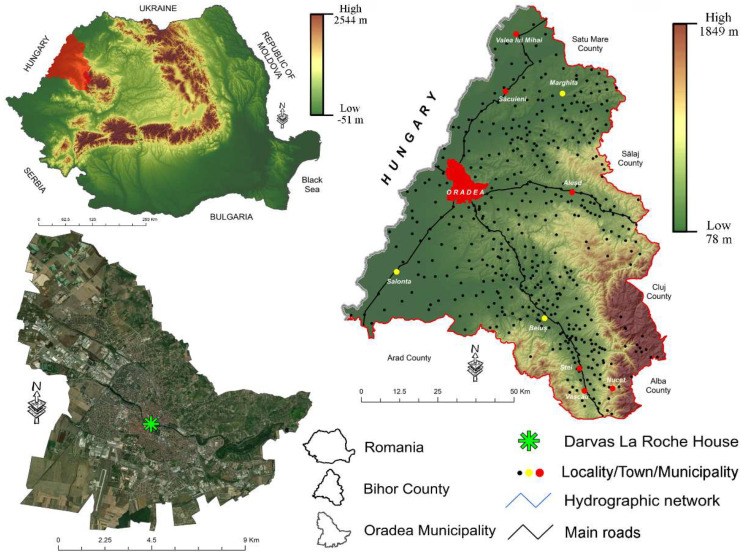
Location of DarvasLa Roche House at the national, county and local level.

Based on the previously indicated, the current study aims to monitor the internal microclimate within a heritage building built in the Art Nouveau style, which currently functions as a museum, in the Municipality of Oradea, Romania (Figure 1). The research aimed to determine the concentrations of 15 indicators of the internal microclimate that have potentially harmful effects on the exhibits in the museum, as well as the health of its employees and visitors. The final goal is to have a solid basis for the implementation of regulations regarding the internal microclimate within the museum, to protect the exhibits and at the same time not limit the visitors’ need for knowledge. The study is even more important because the museum is of great interest both at the level of the Municipality of Oradea and the western part of Romania, being one of the few museums that present a varied range of exhibits belonging to the end of the 19th century and the beginning of the 20th century. At the same time, the museum building has recently gone through extensive renovation and restoration processes, and the measurements also take into account the determination to what extent these interventions have affected the internal microclimate.

**Figure 2:**
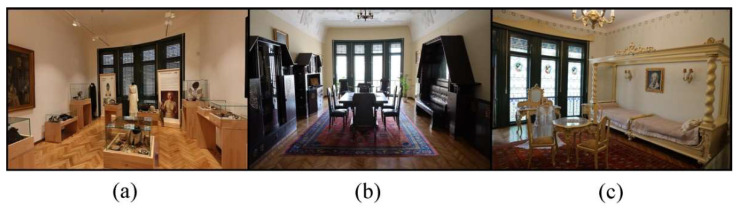
The three rooms within the Darvas-La Roche House that were monitored in order to determine the characteristics of the internal microclimate ((**a**)—Exhibition hall on the ground floor; (**b**)—Great hall; (**c**)—Bedroom).

The determinations were undertaken simultaneously in three rooms of the museum (Figure 2), located in different parts of the building and having various exhibits and properties, the results being comparatively analyzed in order to form an overall picture.

Darvas-La Roche house in Oradea was built between 1911 and 1912 by the brothers László and József Vágó in the Art Nouveau style, adorned with numerous Viennese elements and essential geometrical shapes, white stone plate ribs and ceramic corner studs with metallic enamel, giving it beauty and a distinctive appearance [56,57]. Today, it is one of the most beautiful Art Nouveau museums, which reopened its doors in 2020, after an extensive restoration project to restore it to its former beauty and glory, financed by European funds.

Inside, the period furniture in neo-rococo style is carved from wood painted white. The rooms are furnished with decorative pieces and paintings that define the modern and refined style of life from the beginning of the 20th century [58]. The charming building is unique in its beauty and modernity, being able to stand alongside the most original European architectural creations from the beginning of the 20th century. The building offers an exceptional architectural spectacle, largely preserved in the vision in which it was built more than a hundred years ago.

## 2. Materials and Methods

**Figure 3:**
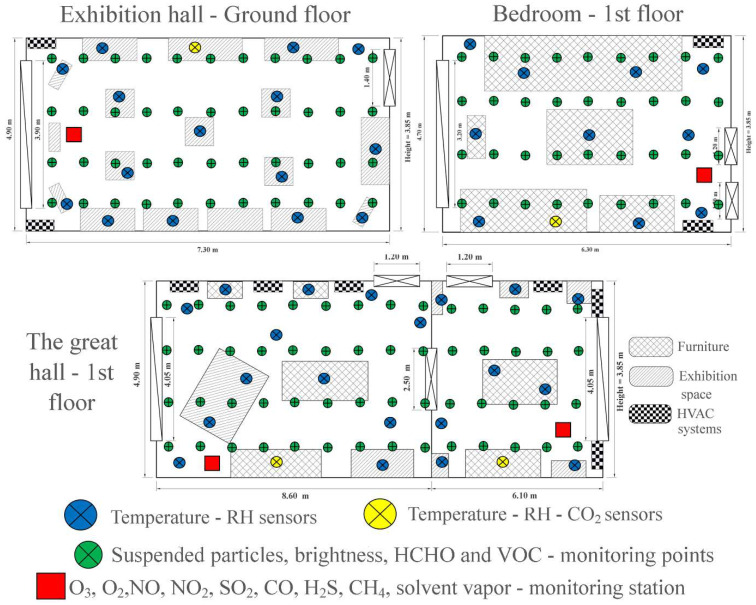
The three rooms analyzed within Darvas-La Roche House, the spatial distribution of sensors and data collection points within them.

The internal microclimate was monitored in three rooms of the museum. These three rooms were carefully chosen so as to present different particularities regarding the nature of the exhibits, the materials they are made of and the way of preservation and display. Thus, the three rooms are some of the most spacious and visited within the museum, containing rare objects from the Belle Époque period (1871–1914) and valuable furniture (Figure 2). The three rooms can be characterized as follows:

- The Exhibition hall–Ground floor of the museum has dimensions of 7.30 m long, 4.90 m wide and 3.85 m high, with a total volume of 137.7 m^3^. It presents to the interested public authentic pieces of clothing, jewelry and various accessories, mostly intended for women. The construction materials of the exhibits are among the most diverse, imposing: wood, leather, textiles, cellulose, stones and precious materials. Unlike other rooms of the museum, most of the exhibits in this room are kept in glass domes, for better preservation. The room communicates with the outside through a door measuring 1.2 m × 2.5 m and a window measuring 3.2 m × 3.9 m;
- The bedroom on the 1st floor of the museum is impressive with dimensions of 6.30 m long, 4.70 m wide and 3.85 m high, with a total volume of approximately 114 m^3^. It is one of the most interesting exhibition halls in the museum, mainly including precious furniture. This room differs from the others in that it is closed to public access, viewing only through the 1.5 m wide door. The room has a window with dimensions of 3.2 × 3.2 m;
- The great hall located on the 1st floor is usually divided into two smaller rooms, which communicate with each other through a 2.5 × 2.5 m door, which is permanently open. For this reason, the two rooms were ventilated as a whole, the internal microclimate not registering very big differences. The two rooms have total dimensions of 14.7 m length, 4.9 m width and 3.85 m height, with a volume of approximately 277.3 m^3^. Within them, you can find especially old furniture and decorations, made of wood or textile materials. They are the rooms where piano concerts, various meetings or workshops take place quite frequently. The two rooms each have a window measuring 4.05 m wide and 2.3 m high (Figure 3).

The determination of the internal microclimate within the three halls under study considered the monitoring of the following parameters: temperature (T), relative humidity (RH), the amount of natural light (NL) and artificial light (AL), concentration of carbon dioxide (CO_2_), suspended particles of 2.5 and 10 µm (PM_2.5_, PM_10_), the concentration of formaldehyde (HCHO), volatile organic compounds (VOC), oxigen concentration (O_2_), sulfur dioxide (SO_2_), ozone (O_3_), nitrogen dioxide (NO_2_) and nitric oxide (NO), hydrogen sulfide (H_2_S), carbon monoxide (CO) and methane (CH_4_) concentration. Data collection was carried out between September 2021 and March 2022, during 28 weeks, and the results were compared with the international standards in force for establishing the air quality inside the museum.

All these indicators were monitored with the help of data logger sensors, which aim to record data automatically. All the sensors were set to capture data at hourly intervals, in order to obtain a database as voluminous as possible. The positioning of the sensors at the level of the rooms was done in such a way as to obtain the best possible coverage of them, leaving at the same time the possibility of performing some analyses of the distribution of these parameters at the level of the entire rooms.

Temperature and RH were mainly monitored with Klimalogg Pro thermo-hygrometers (TFA, Ottersberg, Germany). It allows for monitoring and data recording through 8 individual sensors + collection station. Klimalogg Pro has an accuracy of ±0.1 °C in terms of temperature and ±3% in the case of RH. The temperature and RH were obtained from 16 positions in the Exhibition hall–Ground floor, from 11 positions in the room dedicated to the bedroom, respectively, 22 positions in the great hall on the 1st Floor (Figure 3).

CO_2_ concentration was measured using four Extech SD800 (Extech Instruments, Nashua, NH, USA) datalogger devices. These, in addition to determining the CO_2_ indicator, also include temperature and RH determinants; thus, the data that Extech SD800 provided were also used for the analysis of temperature and RH fluctuations. The accuracy of the device amounts to ±40 ppm in terms of determining the CO_2_ concentration, ±0.8 °C in the case of temperature, and ±4% for determining the relative humidity of the air. CO_2_ concentration was decided to be determined from four points, positioned side-center at the level of the analyzed halls. Thus, an attempt was made to limit the obtaining of erroneous values, determined by the momentary activity of visitors or employees inside the rooms (Figure 3).

For a better determination of indoor air quality and possible negative effects on employees and visitors, all sensors were placed (as far as possible) at the height of a person of average height (approximately 1.7 m).

In order to determine the amount of PM, VOC, HCHO, NL and AL it was considered the individualization of some measurement points distributed uniformly within the rooms, in order to obtain the best possible coverage, leaving at the same time the possibility of generating cartograms of the spatial distribution of the indicators analyze. The data collection points are positioned in four rows approximately 50 cm from each other (Figure 3). Thus, in the Exhibition Hall–Ground Floor, 44 such collection points were established, in the bedroom there were 38 points, while in the two large Halls located on the 1st floor of the museum, the data were revealed from a total of 56 points. The data were collected three times a day, once early in the morning before the museum opens for visitors, once at noon when the activity is at its peak, and the last recording was made in the evening, immediately after the museum closed.

The concentration of suspended particles in the total volume of air was determined using a PCE-PCO 2 handheld device (PCE Instruments UK, Southampton, UK). This device has the property of analyzing air samples taken from the interior and displaying the concentration of suspended particles individually, for those with the size of 0.3 µm, 0.5 µm, 1.0 µm, 2.5 µm, 5.0 µm and 10 µm. In the current study, only particles with a size of 2.5 µm and 10 µm were analyzed. At the same time, the device determines the indicators with an error of up to 5%, allowing for the saving of 5000 sets of data in the internal memory.

VOC and HCHO concentrations were determined using a BLATN BR-smart-123s (BLATN Science & Technology, Beijing, China) device. It determines has measurement ranges between 0–5.0 mg/m^3^ in the case of HCHO and 0–9.9 mg/m^3^ in the case of VOC. The measurement resolution for both indicators is 0.001 mg/m^3^, and the margin of error is up to ±5%.

The brightness, both natural (NL) and artificial (AL), was measured at different times of the day (usually between 9 a.m. and 5 p.m.), using an Extech SDL400 (Extech Instruments, Nashua, USA) luxmeter datalogger. This device renders the amount of light in lux with an accuracy of ±4%.

The test time for the acquisition of the concentrations of the three indicators fell between 30 s and 2 min for each of the points considered, depending on the particularities of each one, the activity within the museum and the results obtained. As in the case of temperature, RH and CO_2_ determination, the test height was preset to 1.7 m.

Regarding the O_2_, O_3_, SO_2_, NO_2_, NO, H_2_S, CO and CH_4_ indicators, their concentrations were determined with the help of an Evikontroll Gas detection and control system (Evikontroll Gas, Tartu, Estonia). Eight sensors from the E2638 series were used, with a precision of 1 ppm (CO and NO), 0.1 ppm (H_2_S, NO_2_ and SO_2_), 0.01 ppm (O_3_) and 0.01% (O_2_ and CH_4_). All the sensors were connected to central stations with a datalogger function to store the measurements taken every 10 min. To determine these pollutants, four measurement positions were used, one each in the exhibition hall on the ground floor and the bedroom, and two in the large hall on the 1st floor, where it was necessary that, for reference to the international standards in force, the pollutant concentrations determined in ppm/m^3^ were transformed into µg/m^3^ or mg/m^3^ taking into account the molar mass of the gas, the ambient temperature and the inside pressure.

## 3. Results

For an adequate preservation of the exhibits and protection of human health, the internal microclimate must be maintained, according to ASHRAE standards [59], at approximately 20 °C (±1–2 °C) in terms of temperature, with daily variations of less than 2 °C. In the case of relative humidity, the ideal value is approximately 50% (±3%), but RH variations in the range of 45–60% are accepted but without very large or very frequent daily variations. A quantity of CO_2_ greater than 1000 ppm [60] can lead to headaches, drowsiness and even breathing problems in case of prolonged exposure [17,61]. Yet, unlike temperature and RH, too much CO_2_ is not recognized as having negative effects on the integrity of the exhibits.

**Figure 4:**
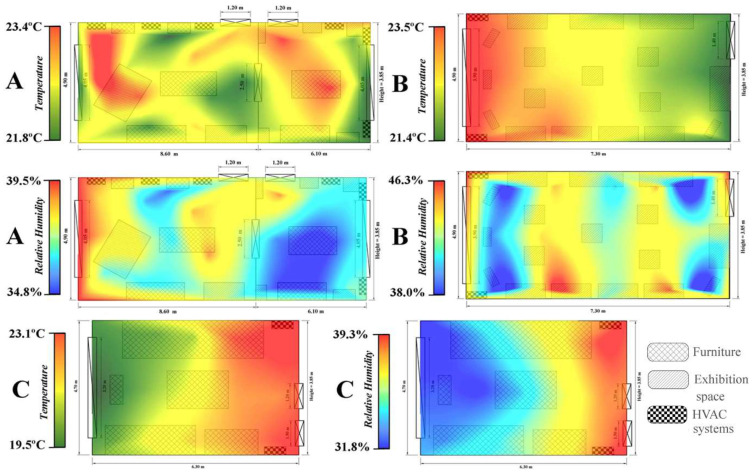
Spatial distribution of temperature and relative humidity within the three analyzed rooms of Darvas-La Roche House ((**A**)—The great hall–1st floor; (**B**)—Exhibition hall–Ground floor; (**C**)—Bedroom–1st floor).

The determinations made in Darvas-La Roche House show that the average temperature in the three analyzed rooms exceeds the accepted standard, with a value of 22.3 °C. At the same time, the average RH value is only 38.3%, approximately 7% lower than the limit allowed by the ASHRAE international standards in force (Figure 4). The only indicator that complies with the standards is the CO_2_ concentration, with an average value of 603.4 ppm, rarely exceeding the upper limit of tolerance set at 1000 ppm.

**Figure 5:**
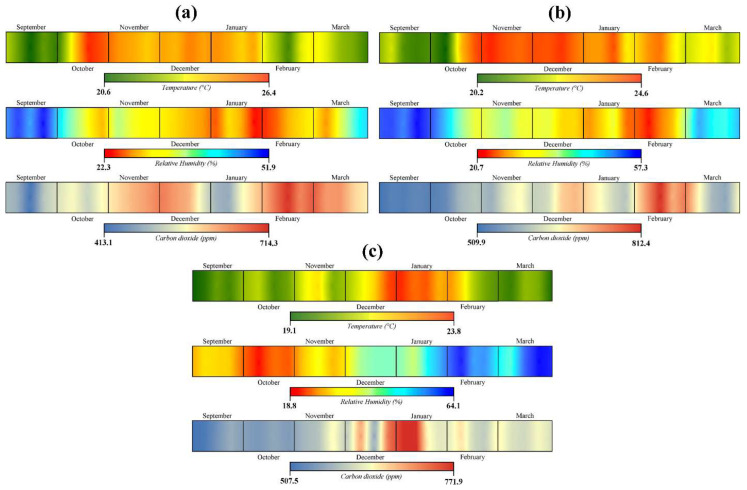
Fluctuations of the average weekly values of temperature (T), relative humidity (RH) and carbon dioxide (CO_2_) concentration ((**a**)—The great hall–1st floor; (**b**)—The exhibition hall–Ground floor; (**c**)—The bedroom–1st floor).

The large exhibition hall on the 1st floor records the highest average temperature value in the analyzed period (22.8 °C), with a maximum amplitude of 8.1 °C, between minimum temperatures of 19.6 °C and maximum temperatures of 27.7 °C. The lowest average weekly values were recorded in the third week of monitoring, being 20.6 °C, while the maximums were recorded in the 6th week and reached 26.4 °C. In 23.9% of the 28 weeks of monitoring, the temperature values fell within the recommended range of 20 °C (±1–2 °C), the rest of the recorded values exceeding the upper limit of 22 °C. The highest values were recorded between October 2021 and January 2022, which indicates the intensive use of HVAC systems for heating the rooms (Figure 5a).

The highest temperatures were recorded, according to Figure 4A, in the center of the small hall dedicated to men, as well as in the north-eastern extremity of the large hall (in this case the high temperatures can be induced by the sun’s rays–considering the immediate vicinity of the window). The distribution of temperatures is still uneven, not taking into account the location of the six HVAC systems within the analyzed halls.

The large hall on the 1st floor records average values of RH during the entire analyzed period of 37.1%. The average weekly values fall between 22.3% in the 19th week and 51.9% in the fourth week. The absolute values are between a minimum of 16% and a maximum of 57%, with a maximum amplitude of 41%. In only 12.8% of the time, the RH values fall within the recommended range of 45–60% and in the rest of the situations the values are lower than 45%. In Figure 5a, the RH values are inversely proportional to those of the temperature in this room of the museum, with the increase in temperature leading to a decrease in RH; thus, the highest RH values are recorded at the beginning and at the end of the monitoring period.

As in the case of the spatial distribution of the temperature, within the large hall on the 1st floor, RH registers an uneven distribution at the level of the hall. The high values are imposed at the extremities and partially in the center of the large hall, while the small values are attributed to the men’s hall (Figure 4A).

Regarding the concentration of CO_2_, the large hall on the 1st floor recorded average values of 580.4 ppm for the entire analyzed period. The average weekly values were comprised between a minimum of 412.8 ppm related to the 3rd week and a maximum of 713.2 ppm recorded in the 22nd week (Figure 5b). The absolute values in the analyzed period were represented by a minimum of 367 ppm and a maximum of 2043 ppm, the amplitude being 1676 ppm.

Only in 0.7% of the cases, the CO_2_ concentration values exceeded the allowed 1000 ppm and in 79% of the time they remained even below the value of 500 ppm.

Regarding the Exhibition hall on the ground floor of the museum, it recorded an average temperature of 22.5 °C during the 28 weeks of monitoring. The average weekly values were between 20.2 °C in the sixth week and 24.6 °C in the ninth week (Figure 5b). The absolute thermal amplitude was 8 °C, with a minimum of 18.7 °C and a maximum of 26.7 °C. In 28.5% of the cases, the temperature values were within the range recommended by the international standards in force; the rest of the values being higher than the allowed. The temperature distribution at the level of the room, presented in Figure 4B, reveals the presence of higher values in the northern half of the exhibition hall, in the area where the two HVAC systems are located; thus, the higher temperatures in that area can be explained.

The Exhibition hall on the ground floor is assigned the highest average values of relative humidity. In the analyzed period, the average of this indicator was 43%, with an absolute minimum of 15% and a maximum of 67%. The weekly averages show the presence of more humid air at the beginning and at the end of the monitoring period, the weekly maximum being recorded in the fourth week, while from October to February, the RH of the air gradually decreases to a minimum of 20.7% related to the 21st week (Figure 5b). In the analyzed period, 27.6% of the obtained values fall within the recommended range of 45–60% RH, 69.7% of them being lower than the ideal, and 3.7% exceeding the upper limit. The spatial distribution of RH values within the room is uneven; the more humid air is concentrated in the middle of the room, while at the extremities and near the window (where the HVAC systems are positioned), the values are lower (Figure 4B).

The exhibition hall on the ground floor also records the highest values in the case of CO_2_ concentration, with an average of 628.3 ppm over the entire monitored period. The average weekly values were comprised between a minimum of 509.8 ppm related to the first week of monitoring and a maximum of 812.4 ppm, recorded in the 22nd week (Figure 5b). The absolute maximum value was 1966 ppm, a concentration potentially harmful to human health, while the absolute minimum was 401 ppm. Only 1.8% of the records obtained show a concentration that exceeds the quality standard of 1000 ppm, in the rest of the cases the CO_2_ values fall within the standards; 72% of the values obtained even showing a concentration below 500 ppm.

The lowest average temperatures were recorded in the bedroom located on the 1st floor of the museum, 21.4 °C being the average of the entire monitored period. The average weekly values were between 19.1 °C in the first week and 23.8 °C in the sixteenth week (Figure 5c). The absolute thermal amplitude was 6.1 °C, with a minimum of 18.7 °C and a maximum of 24.8 °C. In 90.6% of the monitoring period, the temperature values were within the range recommended by the international standards in force, the rest of the values being higher than the allowed ones. The highest temperatures were recorded between mid-December and mid-February, these being attributed to the southern half of the room, where the two HVAC systems are positioned (Figure 4C).

The relative humidity registered an average value of 35.2% during the monitoring period in the bedroom, with an absolute minimum value of 13.5% and an absolute maximum value of 72.2%. The weekly averages show the presence of more humid air in the second half of the monitoring period (starting with December), the maximum weekly average value being recorded in the 27th week (64.1%), while the minimum of 18.8% is attributed to the 6th week of monitoring (Figure 5c). In the analyzed period, 5.1% of the values obtained fall within the recommended range of 45–60% RH, 88.7% of them being lower than the ideal, and 6.2% exceeding the upper limit. The spatial distribution of the RH values within the room indicates an indirect and proportional relationship between this indicator and the temperature, the air being drier in the areas where the temperature is higher, these values increasing with the proximity to the window of the room, where the highest values are recorded (Figure 4C).

The CO_2_ indicator recorded average values of 601.2 ppm for the entire analyzed period, in the bedroom located on the 1st floor of the museum. The average weekly values were comprised between a minimum of 507.5 ppm related to the first week and a maximum of 771.9 ppm recorded in the 17th week (Figure 5c). The absolute values in the analyzed period were represented by a minimum of 456 ppm and a maximum of 2743 ppm, the amplitude being 2287 ppm. In 1.3% of the total data obtained, the values exceed the allowed limit of 1000 ppm, in the rest of the cases the values are below this limit, and in 31.5% of the cases they are even below 500 ppm. The highest values were recorded in February, as well as in the first week of March and the second half of December (Figure 5c).

Fluctuations in temperature, RH and CO_2_ show similarities regarding the Exhibition hall on the 1st floor and the Exhibition hall on the ground floor, both of which record temperatures in accordance with international standards from September to mid-October and from February to March. As for the RH values in the two rooms, they remain within normal limits in September and early November and at the end of March, otherwise they are below the imposed limits (Figure 5a,b).

A particular case is that of the bedroom, where the average values of the compliant temperature are recorded only in November and partially in the December-January period, while the RH has concentrations between 45 and 60% in most of the period between December and March (Figure 5c). In the case of CO_2_, it remains within normal limits throughout the monitoring period, but records an increase in values between January and February 2022, in the case of all three Halls considered.

For the process of deterioration of museum exhibits to take place, energy is needed, and light is one of the most powerful sources of energy [62]. Some exhibits are more fragile than others when exposed to light, such as those made of cellulose and textile materials [63]; light can introduce oxidation, discoloration, increased fragility, loss of elasticity, yellowing or blackening [64,65]. The international standards in force [66] indicate that the quantity of optical radiation must be as far as possible between 50 and 200 lux. Values that exceed this threshold can determine the acceleration of the degradation process of the materials arranged in museums.

During the analyzed period, the three rooms within Darvas-La Roche House recorded an average luminosity value of 161.5 lux. The average value of natural light (NL) was 87.8 lux, while the average for the entire period regarding artificial light (AL) was 235.2 lux. If in terms of NL, the obtained values fall within international standards in force, AL slightly exceeds the limits set by them. Analyzing comparatively the luminosity areas in the three monitored rooms, some dysfunctions can be observed. The high values of NL are concentrated in the immediate vicinity of the large windows of the rooms, while AL is characterized by a random distribution of brightness, depending on the positioning of the light reflectors and the angle of incidence between the light rays and the adjacent surfaces.

**Figure 6:**
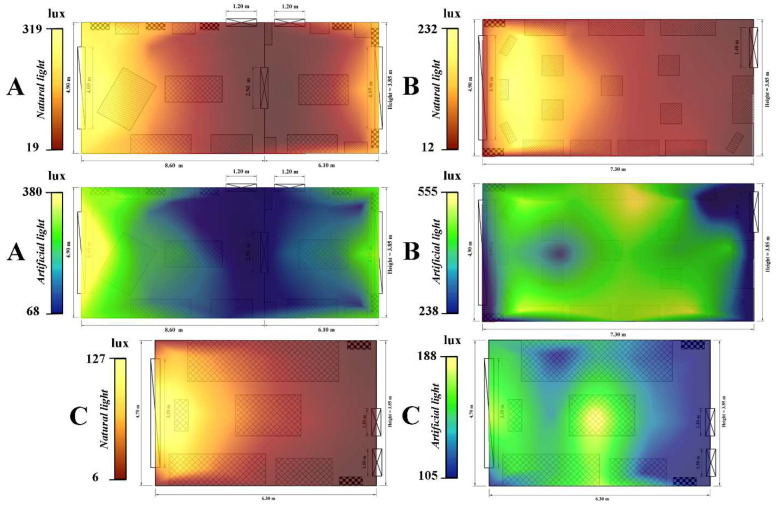
Spatial distribution of brightness (both natural light–NL and artificial light–AL) within the three analyzed rooms of the Darvas-La Roche House ((**A**)—The great hall–1st floor; (**B**)—Exhibition hall–Ground floor; (**C**)—Bedroom–1st floor).

The bedroom of the museum recorded the lowest values during the monitoring. The averages were 34 lux regarding NL and 137 lux in the case of AL. Regarding the average values/data collection point, they vary between a maximum of 127 lux and a minimum of 6 lux in the case of NL and between 189 lux and 105 lux for AL (Figure 6C). The bedroom is followed by the exhibition rooms located on the 1st floor, which recorded average values for the entire period of 132 lux in the case of NL and 175 lux in the case of AL. The average values per collection point indicate that NL varied between 19 lux and 320 lux during the analyzed period, while AL had a minimum value of 68 lux and a maximum of 381 lux (Figure 6B).

A special case is presented by the showroom on the ground floor, where the average values were 76 lux regarding NL and 431 lux for AL. Natural light had average values per data collection point between 12 lux and 232 lux. Very high values were recorded at AL, which varied between a minimum of 238 lux and a maximum of 555 lux (Figure 6A). These high values are due to the fact that in this exhibition hall there are many very powerful light reflectors, which have the role of providing visitors with an easier environment for observing the exhibits; but at the same time, all turned on at once cause the brightness values to increase exponentially.

High concentrations of dust in suspension have harmful effects both for human health and for the preservation of museum exhibits. Due to them, the suspended dust values must be kept as low as possible to maintain a clean environment. Setting precise limits regarding the concentration of dust in suspension is quite difficult, considering that the nature of the particles and their physical and chemical characteristics must be taken into account, as well as the exposure time of people and objects. According to EPA Standards regarding the quality of the surrounding air, the thresholds allowed for suspended particles are 12 µg/m^3^ multi-year average and up to 35 µg/m^3^ daily variation allowed [67]. To maintain human health, the concentration of particles in suspension must not exceed 15 µg/m^3^; keeping values under such a concentration denotes a clean environment, conducive to human activity [7,68,69].

Depending on the aerodynamics of suspended particles, they are usually divided into two large categories: coarse particles (PM_10_) of 10 μm and fine particles (PM_2.5_), with a size of 2.5 μm [67]. The two categories were the object of the determinations and analyzes of the current study.

The three rooms analyzed within Darvas-La Roche House during the determinations, recorded an average value of suspended particles of 19.2 µg/m^3^, of which PM_2.5_ stands out with 7.1 µg/m^3^, while PM_10_ had a value average of 12.1 µg/m^3^.

**Figure 7:**
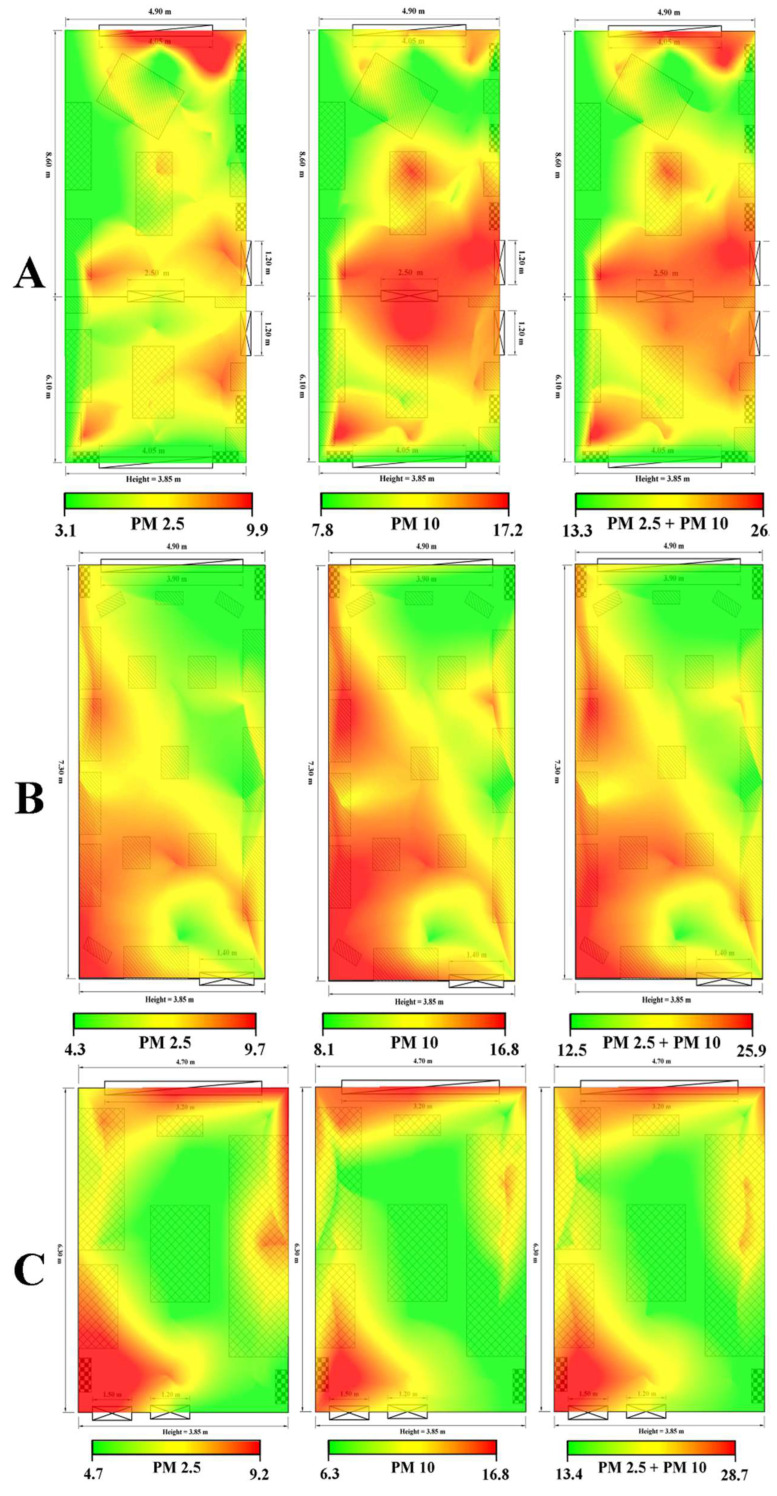
Spatial distribution of suspended particles (PM_2.5_, PM_10_ and their combination) within the three analyzed rooms of Darvas-La Roche House, determined in µg/m^3^ ((**A**)—The great hall–1st floor; (**B**)—Exhibition hall–Ground floor; (**C**)—Bedroom–1st floor).

The exhibition halls on the 1st floor of the museum registered an average value of suspended particles of 18.7 µg/m^3^ (PM_2.5_—6.8 µg/m^3^; PM_10_—11.9 µg/m^3^). The minimum values being 4 µg/m^3^ (PM_2.5_—1 µg/m^3^; PM_10_—3 µg/m^3^), while the maximum values are 45 µg/m^3^ (PM_2.5_—16 µg/m^3^; PM_10_—29 µg/m^3^). The high values are concentrated in the immediate vicinity of the two entrances to the rooms, extending to their center, as well as near the windows (Figure 7A).

The exhibition halls on the ground floor record the lowest values of the concentration of suspended particles, with an average of 18.2 µg/m^3^, of which PM_2.5_ recorded an average of 6.7 µg/m^3^, and PM_10_ had 11.5 µg/m^3^. The minimum values in this room are 7 µg/m^3^ (PM_2.5_—2 µg/m^3^; PM_10_—5 µg/m^3^), while the maximums reach 53 µg/m^3^ (PM_2.5_—19 µg/m^3^; PM_10_—34 µg/m^3^). According to Figure 7B, the high concentrations of suspended particles are concentrated in the western half of the room, which can be explained by the fact that it is a route frequented by tourists, due to the attractiveness of the exhibits in the respective area.

The bedroom hall represents the area with the highest load of suspended particles in the air among all the analyzed rooms. The average value for the entire period here reaches 19.9 µg/m^3^, with 7.9 µg/m^3^ average of PM_2.5_ and 12.9 µg/m^3^ average of PM_10_. The absolute minimum values in the bedroom were 7 µg/m^3^ (PM_2.5_—2 µg/m^3^; PM_10_—5 µg/m^3^), and the maximums indicate no less than 53 µg/m^3^ (PM_2.5_—21 µg/m^3^; PM_10_—32 µg/m^3^). High concentrations of dust in suspension are evident (Figure 6C) mainly in the vicinity of the open door from which visitors look at the restricted room, thus picking up the fine dust particles. High values were also recorded near the window, the intense traffic on the street leading to the lifting of dust in the air and its penetration inside the room.

As in the case of suspended particles, volatile organic compounds (VOC) and formaldehyde (HCHO) can cause negative effects on human health, even at fairly low concentrations and a limited exposure time [38,70]. In the case of VOC, the World Health Organization (WHO) within the Air quality guidelines for Europe [71] set the acceptability thresholds of this indicator at 1 mg/m^3^. Higher values are potentially harmful to human health, and exposure to a concentration higher than 3 mg/m^3^ over a prolonged period has recognized negative effects on human health [7]. Volatile organic compounds (VOC) recorded high values in the analyzed period, above the threshold of 3 mg/m^3^ regulated by international standards. The average of this indicator was 4.17 mg/m^3^, approximately 73.8% of the measurements performed exceeding the allowed thresholds.

**Figure 8:**
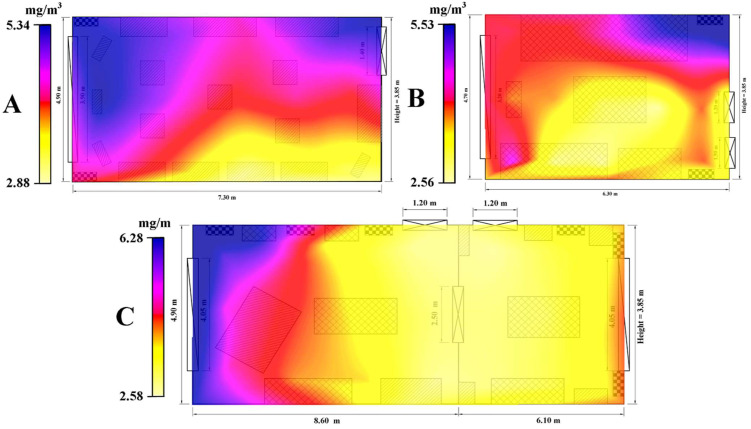
Spatial distribution of the concentration of volatile organic compounds (VOC) in the three analyzed rooms of Darvas-La Roche House–taking into account the average of each data collection position for the entire measured period ((**A**)—Exhibition hall–Ground floor; (**B**)—Bedroom–1st floor; (**C**)—The great hall–1st floor).

The lowest VOC values were recorded in the exhibition halls located on the 1st floor of the museum; the average being 3.84 mg/m^3^, with absolute values between a minimum of 1.16 mg/m^3^ and a maximum of 8.23 mg/m^3^. This was followed by the bedroom hall with an average of 4.23 mg/m^3^ (1.33 mg/m^3^ absolute minimum and 9.12 mg/m^3^ absolute maximum) and the exhibition hall on the ground floor, which recorded the highest average value, 4.44 mg/m^3^ (1.83 mg/m^3^ absolute minimum and 10.18 mg/m^3^ absolute maximum). Figure 8 indicates a differentiated distribution of the average (per data collection point) of volatile organic compounds within the three studied areas of the museum, but having a common characteristic, the high values of VOC being registered near the HVAC systems and the areas where very old furniture objects are exposed. These two components can represent the main sources of VOCs in the museum, associated with the proximity to the windows, where the temperature and humidity are higher due to external factors, which can lead to the easier release of volatile components.

As for formaldehyde concentration (HCHO), The Agency for Toxic Substances and Disease Registry (ATSDR), quoted by United States Environmental Protection Agency (EPA) [72], indicated the value of 0.004 mg/m^3^ as a minimum dangerous value on human health. If the HCHO values are maintained up to this threshold, the risk of illness is non-existent, even with regular exposure. If the concentrations of this indicator exceed the allowed value, different conditions may appear such as cough, sore throat, nausea, eye and respiratory system irritations, asthmatic attacks and even cancer. People with allergies and low immunity are especially targeted, such as children, the elderly or people with respiratory problems [73,74].

**Figure 9:**
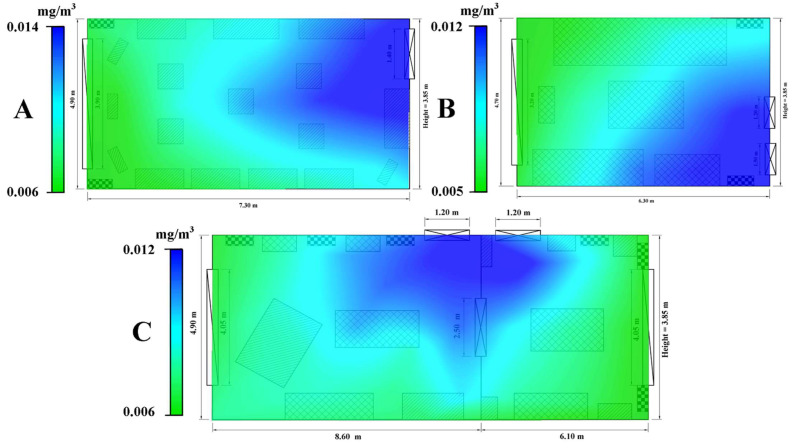
Spatial distribution of formaldehyde (HCHO) concentration values in the three analyzed rooms of Darvas-La Roche House–taking into account the average of each data collection position for the entire measured period ((**A**)—Exhibition hall–Ground floor; (**B**)—Bedroom–1st floor; (**C**)—The great hall–1st floor).

The average of the three rooms in terms of HCHO concentration was 0.009 mg/m^3^. The exhibition hall on the ground floor recorded an average of 0.011 mg/m^3^, the averages of the collection points varying between 0.008 mg/m^3^ and 0.013 mg/m^3^ (Figure 9A). The absolute values varied between a minimum of 0.006 mg/m^3^ and 0.019 mg/m^3^. Both in the case of the bedroom and in the case of the exhibition rooms on the 1st floor, the concentration of HCHO recorded an average value of 0.008 mg/m^3^. The absolute values are found between 0.005 mg/m^3^ and 0.019 mg/m^3^ (Figure 9C) in the case of the rooms on the first floor, with 0.006 mg/m^3^ and 0.021 mg/m^3^ for the bedroom (Figure 9B). According to Figure 9, high concentrations of HCHO are identified near the entrances of the monitored rooms, the values decreasing with the proximity to their extremities, with minimums in the area of the windows.

Even if HCHO values exceed the thresholds allowed by international standards, the relatively low concentrations and the limited exposure of employees and visitors make the negative effects of HCHO on human health all remain low.

**Figure 10:**
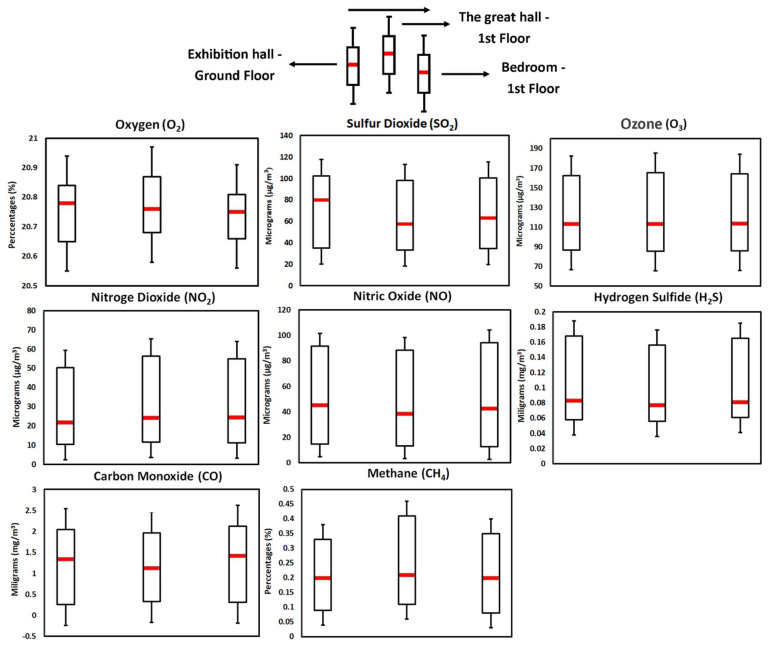
The minimum, maximum and average concentrations of O_2_, SO_2_, O_3_, NO_2_, NO, H_2_S, CO and CH_4_ in the three monitored rooms of the Darvas-La Roche House.

Oxygen is a critical component in the process of cellular respiration, therefore the human body (especially the brain) needs an optimal concentration of O_2_ in the air. Regarding the allowed O_2_ concentrations, they fall between 19.5% and 23.5%, according to Environmental Indoor Air Quality Testing Consulting [75]. A prolonged exposure to values that exceed the mentioned thresholds can lead to tachycardia, thinking and coordination disorders, exhaustion, nausea, vomiting, etc. [76]. In the case of Darvas-La Roche House, the O_2_ recorded an average value over the entire period of 20.76% (20.78%—ground floor exhibition hall; 20.76%—great hall; 20.75%—bedroom). At the same time, the evolution of this indicator is linear in all three rooms, fluctuating within a gap of only 0.22%, absolute values being a maximum of 20.87% and a minimum of 20.65% (Figure 10). All these values fall within the international standards in force, representing a favorable environment for human activity.

According to the World Health Organization [77], the concentration of sulfur dioxide (SO_2_) in indoor environments must not exceed 50 µg/m^3^ in terms of the multi-year average and 125 µg/m3 in the case of the daily average. Concentrations above these limits can lead to reduced respiratory capacity and breathing difficulties [78,79]; the most prone people being those who suffer from asthma [80,81]. The average concentration of SO_2_ in the case of the present study was 66.9 µg/m^3^, values higher than the thresholds regulated by means of the international standards in force. The lowest average values were recorded in the great hall (57.7 µg/m^3^), followed by the bedroom (63.3 µg/m^3^) and the exhibition hall on the ground floor (79.8 µg/m^3^). The absolute values were between 102.6 µg/m^3^ (in the ground floor exhibition hall) and 33.4 µg/m^3^ (in the large hall) (Figure 10). Thus, up to 25% of the allowed annual thresholds are exceeded, while the daily thresholds were not exceeded in any situation during the monitoring period.

Ozone (O_3_), as well as other photochemical oxidants, is formed by the action of short-wavelength radiation on NO_2_. High amounts of VOCs favor the process and lead to the appearance of high concentrations of O_3_ [82,83]. WHO [77] indicates the value of 120 µg/m^3^ as the limit allowed for exposure for 8 h/day, for minimal health effects. O_3_ toxicity has the potential to manifest itself when that limit is exceeded and exposure to that environment continues for a long time. The determinations carried out in Darvas-La Roche House indicate average concentrations of 113.4 µg/m^3^ for the entire analyzed period (113.2 µg/m^3^—ground floor exhibition hall; 113.4 µg/m^3^—great hall; 113.7 µg/m^3^—bedroom) (Figure 9). The maximum values were in the range of 162.3–165.3 µg/m^3^ but only for very short periods of time, without representing a danger to human health. In contrast, the minimum values of O_3_ concentrations were between 86.8 µg/m^3^ and 86.6 µg/m^3^.

The main source of NO and NO_2_ is combustion and especially internal combustion engines [84]. They are toxic gases for human health if found in too high concentrations; studies in the field associate high values of NO_2_ with cardiovascular diseases [85,86,87,88] and respiratory diseases [89,90,91,92]. The occurrence of adverse effects largely depends on the concentration of the pollutant, the exposure time and pre-existing conditions, therefore it is quite difficult to identify a threshold regarding the allowed concentration of these pollutants. According to WHO [77], it is recommended that both NO and NO_2_ concentrations do not exceed the value of 40 µg/m^3^ as regards the annual average, respectively, 75 µg/m^3^ for a short-term exposure. The determinations made within the museum indicate an average concentration of NO of 45.2 µg/m^3^ and NO_2_ of 21 µg/m^3^. The highest values of NO were recorded in the exhibition hall on the ground floor (91.5 µg/m^3^) and in the bedroom (92.2 µg/m^3^), while the minimum values indicate 13.3 µg/m^3^ in the large hall and 12.8 µg /m^3^ in the bedroom. Regarding NO_2_, the absolute maximum values reach 56.2 µg/m^3^ in the great hall and 54.8 µg/m^3^ in the bedroom, and the absolute minimum values were 10.3 µg/m^3^ in the exhibition hall on the ground floor, 11.2 µg/m^3^ in the bedroom and 11.5 µg/m^3^ in the large hall (Figure 10).

Among the first notable effects of hydrogen sulfide (H_2_S) in the indoor environment, at low concentrations, are unpleasant odors [93]. Little is known about the thresholds that, once exceeded, induce diseases on the human body, but the lowest level of H_2_S concentration at which negative effects have been reported is 15 mg/m^3^ (strong eye irritation). In addition to eye irritation, different concentrations of the pollutant can cause vision problems, pulmonary edema, strong central nervous system stimulation, etc. [94,95,96,97]. The guideline [77] regarding the acceptable concentration of H_2_S is approximately 0.15 mg/m^3^ daily average, but exposure to concentrations of up to 1.5 mg/m^3^ is unlikely to cause health problems. The measured values of this pollutant amount to an average of 0.083 mg/m^3^ for the entire analyzed period, with absolute maximum values of 0.168 mg/m^3^ (exhibition hall on the ground floor) and absolute minimum values of 0.056 mg/m^3^ (large hall) (Figure 10).

Carbon monoxide (CO) diffuses quickly in alveolar, capillary and placental membranes, binding to hemoglobin and forming carboxyhemoglobin (COHb), which is a specific biomarker of blood exposure. COHb reduces the oxygen transport capacity in the blood and affects its release in the extravascular tissues [98]. This can lead, at certain concentrations, to coordination disorders, concentration disorders and cognitive performance [99,100,101], while high concentrations of CO can even lead to cardiovascular diseases and myocardial infarction [102,103]. The World Health Organization [77] regulates the concentrations of CO considered dangerous for human health at 10 mg/m^3^ for an exposure of 8 h per day and 30 mg/m^3^ for an exposure of 1 h per day. Comparing the values measured in Darvas-La Roche House to these standards, indicates that there is no exceedance of the allowed concentrations during the study period. Thus, the average value of the CO concentration was 1.34 mg/m^3^ (1.48 mg/m^3^ in the exhibition hall on the ground floor; 1.15 mg/m^3^ in the great hall; 1.4 mg/m^3^ in the bedroom). The maximum absolute values amounted to 2.12 mg/m^3^ (in the bedroom), and the minimum values to 0.26 mg/m^3^ (in the great hall) (Figure 10).

High concentrations of methane (CH_4_) can cause mood swings, slurred speech, vision problems, memory loss, nausea, vomiting, flushing and headaches [104]. It is an asphyxiating gas, which can replace O_2_ inside the rooms. There are no unanimously accepted limits in the workplace or in homes in the case of CH_4_, but it can be the basis of a content lower than 15–18% in terms of O_2_, something that has the potential to harm human health [105,106]. It is indicated that the concentration of CH_4_ be kept below 5%, considering on the one hand that CH_4_ is flammable at concentrations between 5% and 15%, but also due to the fact that CH_4_ can potentiate the negative effects on the human health of other pollutants present in the air [107,108]. The values determined in the present study are at 0.20% with regard to the average CH_4_. The highest values amount to approximately 0.41% in the great hall, 0.35% in the exhibition hall on the side and 0.33% in the bedroom (Figure 10). The absolute minimum values of CH_4_ do not exceed 0.10% (0.09% in the main hall and the exhibition hall on the ground floor; 0.08% in the bedroom).

In order to summarize the quality of the internal microclimate in the three analyzed rooms, it was considered to create an index based on the fluctuations of a 15 indicators per day, taking into account the international standards in force for each indicator. Thus, the number of days in which the value of the indicators fall within the rules in force for the protection of exhibits and for the comfort/health of employees and visitors was calculated: 18–22 °C in terms of temperature, 45–60% in the case of RH [59], below 1000 ppm for the amount of CO_2_ [60], up to 200 lux in term of brightness [66], maximum 12 µg/m^3^ in the case of suspended particles [67], up to 3 mg/m^3^ concentration of VOC [71], below 0.004 mg/m^3^ concentration of HCHO [72], between 19.5% and 23.5% in terms of O_2_ concentration [75], below 50 µg/m^3^ SO_2_ concentration [77], below 120 µg/m^3^ in term of O_3_ concentration [77], less than 40 µg/m^3^ both in as regards NO, as well as NO_2_ [77], below 0.15 mg/m^3^ concentration of H_2_S [77], below 10 mg/m^3^ concentration of CO and less than 5% CH_4_ in indoor air [103].

**Figure 11:**
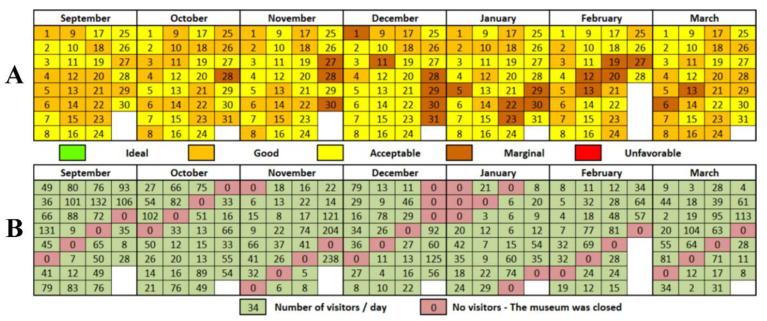
The quality of indoor microclimate expressed on each of the 212 monitoring days ((**A**)—the days in which the values of the 15 indicators recorded ideal/good/acceptable/marginal/unfavorable conditions according to the standards in force; (**B**)—the number of visitors registered for each of the internal microclimate monitoring days).

For the effective composition of the index, the average of each indicator was calculated for each day out of the 212 monitored, taking into account all three rooms, these being grouped within five quality levels, as follows:

Ideal—all 15 indicators of the internal microclimate recorded on the respective day have values that fall within the international standards in force;

Good—≥10 indicators of the internal microclimate recorded on the respective day have values that fall within the international standards in force;

Acceptable—7–9 indicators of the internal microclimate recorded on the respective day have values that fall within the international standards in force;

Marginal—4–6 indicators of the internal microclimate recorded on the respective day have values that fall within the international standards in force;

Unfavorable—<4 indicators of the internal microclimate recorded on the respective day have values that fit the international standards in force (Figure 11).

According to the analysis presented in Figure 11A, it shows that there is no situation in which the conditions of the internal microclimate register values in accordance with the standards for all 15 indicators on the same day; thus, the ideal conditions are not met on any day of the analyzed period. This is due to the fact that there are several indicators that were always outside the range recommended by international standards. First of all, it is about VOC and HCHO, which recorded a lot of exceedances, but also T and RH, which fluctuated quite a lot in the analyzed period. The four indicators make ideal conditions regarding the internal microclimate impossible to achieve in the analyzed period.

The good conditions define 79 days (37.3% of the total), being relatively evenly distributed in the analyzed period, but with peaks in terms of the number of days in the autumn (September-October) and spring (March) periods. This indicator was obtained every time following the association of optimal values of pollutants (O_2_, O_3_, CO_2_, SO_2_, NO, NO_2_, H_2_S, CO, CH_4_) with values that do not exceed the accepted standards for physical factors of the microclimate (T, RH and PM).

Acceptable conditions for the conservation of exhibits and the development of human activities is the most common indicator and have narrowed in 111 days (52.4% of the total). It was most often found in the winter period (between November and February), when the good indicator is numerically narrower. Most of the time, it was obtained by combining the exceedances of the VOC and HCHO indicators with those of T, RH and PM, less often with CO_2_, SO_2_, O_3_, NO and brightness.

A moderate risk of damage to the exhibits and damage to human health was recorded in 22 days (10.3% of the total), when the quality of the internal microclimate was categorized as marginal. Such an indicator was concretized by associating (most of the time) the optimal values of brightness, O_2_, NO_2_, NO, H_2_S, CO and CH_4_; the rest of the indicators registering exceedances of international standards on the respective days. December (6 days), January and February (5 days each) are the months in which the internal microclimate was most often categorized as marginal (Figure 11A).

An unfavorable indicator was not recorded in any of the monitoring days. This is mainly due to the fact that O_2_, CO and CH_4_ did not register any exceedances of international standards during the monitoring period, while H_2_S, NO_2_ and NO had only sporadic exceedances, insignificant most of the time to influence daily average.

A comparative analysis of Figure 11A,B indicates that the human component (number of visitors/day) is also a determining factor of the internal microclimate in Darvas-La Roche House. Therefore, in the 34 days in which the museum was closed to public access during the analyzed period, the internal microclimate was classified most of the time as good (24 days) or acceptable (11 days) (Figure 11B). In these situations, up to 13 indicators recorded values that fell within the international standards to which they were reported. The second situation considers the days when the internal microclimate was categorized as marginal. In these situations, influxes of tourists can be identified; 1875 people out of a total of 7060 visited the museum in the 22 days in which marginal conditions were recorded. This means a percentage of 26.6% of the total number of visits and an average of 85.2 visitors/day, given that in the 111 days with acceptable conditions, 45.3% of the total number of visits (3219 visitors) and an average of 29 visitors/day were recorded, and in the days with good conditions (79 days), 28.1% of the total visitors (1984 visitors) were present in the museum with an average of 25.1 visitors/day.

## 4. Discussion

The analysis shows that most days with improper microclimate conditions are identified in the winter months, when HVAC systems are most frequently used. These systems cause the temperature inside to rise most of the time above 23 or 24 °C (with peaks close to 28 °C), while the RH registers values even lower than 30% (sometimes below 20%). The spatial distribution of T and RH indicates very high values of both indicators in the immediate vicinity of the HVAC systems, the values decreasing with their distance.

At the same time, excessive ventilation associated with high temperature causes PM to be dislodged, so that during this period the peaks are individualized in terms of PM_2.5_ and PM_10_. Zhang et al. [109] and Wang et al. [110] attribute the high concentrations of PM in the indoor air to the high temperature, the combined influence of these factors further determining a high incidence of cardiovascular and respiratory diseases; the effects of PM on human health largely depend on temperature. The high concentration of PM in the air is mainly due to visitors, who bring dust from the outside environment and displace that already deposited [111], something that can also be observed on the spatial distribution of PM within the three analyzed halls; the highest values being recorded on sightseeing routes, while days with numerous tourists recorded peaks in terms of the concentration of PM in the air. At the same time, the values above the allowed limit of this indicator can also originate in the external environment. In Figure 7, it can be seen that high values of both PM_2.5_ and PM_10_ are also recorded in the immediate vicinity of the windows, which face one of the busiest streets in the city. Thus, the influence of the street can have an important effect on the concentration of PM in the indoor air, taking also into account the fact that often the windows are left open for ventilation and the stained-glass windows do not offer perfect insulation against suspended particles.

The external environment also leaves its mark on the concentrations of gaseous pollutants in the internal environment, as shown, among others, in the studies of Uring et al. [5], Cincinelli et al. [26], Schieweck and Salthammer [37] or Hu et al. [112]. Thus, one of the main sources of SO_2_, NO_2_, NO, H_2_S, CO, O_3_ and CH_4_ for Darvas-La Roche House is definitely the outside environment. This is more relevant since the vast majority of these pollutants are a result of burning fossil fuels [80], and the museum is located on a street heavily traveled by cars, while others may have their origin inside the museum, being determined by the chemical reactions that form between different pollutants (may be the case of O_3_), or by different improper installations that aim to purify and/or disinfect the air (O_3_ and H_2_S).

An aspect to be taken into account for identifying the causes underlying the values above the allowed limit of pollutants is the fact that the museum was recently renovated. The US States Environmental Protection Agency [113] and Kristak et al. [114] states that new construction materials, especially insulating materials, paints, varnishes, adhesives and also finished wood composites represent one of the most important sources of gaseous pollutants in indoor environments. These materials are more prone to the elimination of VOCs (implicitly HCHO) when they are associated with high values of T and low RH, as identified by Tran et al. [115] and Fang et al. [116] in their studies. The high values of T can also cause VOC and HCHO present in the substances for preserving exhibits, detergents and disinfectants to evaporate and be found in the indoor air in the form of vapors.

Although there have been violations of the international standards in force in 12 of the 15 monitored indicators, in most cases, the violations are quite small and only sporadic. Thus, for SO_2_ and PM, up to 30% of the total values obtained exceeded the allowed thresholds, in the case of O_3_, CO_2_ and NO the percentage was up to 20%, NO_2_ and H_2_S recorded exceedances in less than 3% of cases. Constant exceedances are recorded for T, RH, VOC and HCHO, but still the values of the exceedances are not very high (with the exception of HCHO, which has values 225% higher than the allowed limits) so as to present an imminent danger. Some sporadic exceedances were also recorded in terms of brightness but especially in the case of AL. This component presents less stress on the exhibits than NL, due to the fact that light reflectors inside radiate light of certain colors, while the spectral composition of the natural light is always changing [117].

However, the presence inside the museum of different pollutants leaves the possibility for them to react with each other and thus cause damage to the exhibits, as Pavlogeorgatos also states [118]. The sudden fluctuations of the T and RH values recorded inside can also be considered destructive phenomena on the exhibits, considering the fact that for the best preservation of the materials, in addition to keeping these indicators within the required standards, a constant evolution is also necessary over time, without large fluctuations [119].

As far as human health is concerned, the microclimatic conditions inside the museum have quite limited effects on visitors, taking into account that they usually spend up to two hours inside [28,29]. Abelsohn and Stieb [120] mention the fact that the effects of short-term exposure can only be found in exacerbating the manifestations of pre-existing respiratory and cardiovascular diseases, in no case in determining the appearance of new diseases. The conditions of the internal microclimate can have a more significant impact on the health of the employees, considering the fact that they spend 8 h a day/7 days a week in this environment. Jones [121] and Rahman et al. [122] indicate that employees may feel discomfort (headaches, dizziness, eye irritation, etc.) if the concentrations of pollutants are low; however, if the exposure to them is prolonged or if there are numerous pollutants in the indoor environment, their effect on health is combined. These represent discomforts that usually disappear when leaving the indoor environment.

An additional stress can be introduced both regarding the conservation of artifacts and human health by the combined action of indoor pollutants and microbiological factors (bacteria and fungi) [123,124]. Recent studies in the field [125,126,127] associate non-compliant values of internal microclimate indicators with the proliferation of bacteriological microflora in the air and on objects. This is all the more obvious in the situation where T is very high but also when it, together with RH, fluctuates in a wide spectrum in a very short time [125].

## 5. Conclusions

Monitoring the environmental conditions inside Darvas-La Roche House is of great importance for preserving the exhibits in the best possible conditions and ensuring a clean environment for staff and visitors. The determinations carried out inside show a problematic environment regarding the very frequent fluctuations of the values of the main indicators: T, RH, CO_2_ and PM. Thus, even if T and RH only recorded small exceedances of the international standards in force, the fluctuations were often quite large during the seven months of monitoring, which can induce instability within the exhibits. Exceedings were also recorded for NL and AL, SO_2_, O_3_, NO_2_, NO and H_2_S indicators; yet, these are small enough and still remain, most of the time (over 70% of the determinations), within the limits imposed by international standards, while ideal values were identified regarding the concentrations of O_2_, CO and CH_4_, which never exceeded the allowed thresholds.

Factors with potential hazard on both exhibits and human health were identified in the case of VOC and HCHO, which are individualized by concentrations of 28% (VOC) and 125% (HCHO) higher than those allowed. These are factors that can induce stress, even if the other pollutants are kept at optimal parameters. At the same time, the combined action of high T and low RH values, with high concentrations of HCHO, VOC and PM, can lead to the acceleration of the deterioration of exhibits, as well as the induction of discomfort in people and/or the exacerbation of already existing conditions.

A sharp decrease in indoor microclimatic conditions was identified during the winter, when 22 days recorded a marginal index in terms of air quality. This is largely akin to the improper use of HVAC systems, which cause substantial increases in T and decreases in RH, which can further lead to the removal of a large volume of gaseous pollutants from various materials and the greater displacement of PM. At the same time, the quality of the indoor microclimate was influenced in this period and by the fact that most activities are organized indoors, unlike the spring-autumn period, when they can also be organized in the garden; this can be seen both in the higher concentrations of PM and CO_2_, as well as in the reduction of the amount of O_2_.

In this case, preventive conservation is required, through continuous monitoring and the identification of safe methods to maintain the artifacts in the best possible shape, but without limiting the access of tourists inside. A more rigorous control over the T value is required, especially during the winter period, when the RH must be balanced by humidifiers, to avoid values below the permitted threshold. For this, T and RH sensors can be used that emit alarm signals when the values exceed a set limit. As far as pollutants are concerned, it is necessary, on the one hand, to use installations for air purification, and on the other hand, continuous research is required in order to accurately determine their source and origin. At the same time, as mentioned by Schito et al. [128], in order to set an ideal of conservation and its implementation with the help of HVAC systems, it is necessary to take into account all the exhibits disposed within the museum because each object has particular needs in terms of conservation and internal microclimate parameters.

## 6. Future Work

This paper is part of a multidisciplinary and multidimensional study in order to determine the quality of the internal microclimate in Darvas-La Roche House. The extensive study aims at molecular determinations on the bacteriological and fungal microflora in the air and on the surface of exhibits in order to determine its implications on human health and the integrity of the exhibits.

## Data Availability

The data presented in this study may be obtained on request from the corresponding author.
